# The Introduction of Impella 5.5 in Cardiogenic Shock: A Single-Center, Retrospective Propensity Score-Matched Analysis

**DOI:** 10.3390/jcm14217552

**Published:** 2025-10-24

**Authors:** Maciej Bochenek, Mateusz Sokolski, Anna Kędziora, Barbara Barteczko-Grajek, Grzegorz Bielicki, Kinga Kosiorowska, Maciej Rachwalik, Rafał Nowicki, Michał Kosowski, Magdalena Cielecka, Michał Zakliczyński, Wiktor Kuliczkowski, Roman Przybylski

**Affiliations:** 1 Clinical Department of Heart Transplantation and Mechanical Circulatory Support, Department of Cardiac Surgery and Heart Transplantation, Institute of Heart Diseases, Faculty of Medicine, Wroclaw Medical University, 50-556 Wroclaw, Poland; matsok@gmail.com (M.S.); magdalena.cielecka@usk.wroc.pl (M.C.); michal.zakliczynski@umw.edu.pl (M.Z.); 2 Clinical Department of Cardiac Surgery, Department of Cardiac Surgery and Heart Transplantation, Institute of Heart Diseases, Faculty of Medicine, Wroclaw Medical University, 50-556 Wroclaw, Poland; kdzra.a@gmail.com (A.K.); grzegorz.bielicki@gmail.com (G.B.); kosiorowska.kinga@gmail.com (K.K.); maciej.rachwalik@umw.edu.pl (M.R.); rafal.nowicki@umw.edu.pl (R.N.); 3 Clinical Department of Anaesthesiology and Intensive Therapy, Faculty of Medicine, Wroclaw Medical University, 50-556 Wroclaw, Poland; barbara.barteczko-grajek@umw.edu.pl; 4 Clinical Department of Cardiology, Department of Cardiology, Institute of Heart Diseases, Faculty of Medicine, Wroclaw Medical University, 50-556 Wroclaw, Poland; michal.kosowski@umw.edu.pl (M.K.); wiktor.kuliczkowski@umw.edu.pl (W.K.); 5 Department of Cardiac Surgery and Heart Transplantation, Institute of Heart Diseases, Faculty of Medicine, Wroclaw Medical University, 50-556 Wroclaw, Poland; roman.przybylski@umw.edu.pl

**Keywords:** cardiogenic shock, Impella 5.5, mechanical circulatory support, survival

## Abstract

**Background/Objectives:** Impella 5.5 provides a higher flow rate than smaller microaxial pumps and has been increasingly adopted for cardiogenic shock (CS). This study aimed to evaluate whether its introduction into our Shock Team program in 2023 improved outcomes compared with a historical cohort supported with other mechanical circulatory support (MCS) devices. **Methods:** We retrospectively analyzed patients with CS treated with MCS between 2020 and 2024 at a tertiary center. The Impella 5.5 group (n = 17) included patients managed after device implementation, either as stand-alone or sequential therapy. The historical cohort comprised 40 patients treated with ECMO, Impella CP, CentriMag, or IABP prior to 2023. Propensity score matching (age, sex, etiology, lactate, SCAI stage) generated 17 matched pairs. The primary outcome was survival at discharge, 30 days, 3 months, and 6 months. Secondary outcomes included bridging to recovery, heart transplantation (HTx), durable LVAD, and major complications. **Results**: Impella 5.5 was associated with higher survival at discharge (94.1% vs. 58.8%, *p* = 0.039), 30 days (94.1% vs. 58.8%, *p* = 0.039), and 3 months (94.1% vs. 58.8%, *p* = 0.039). At 6 months, survival remained higher (88.2% vs. 58.8%) but did not reach statistical significance in point analysis (*p* = 0.118). Bridging occurred more frequently with Impella 5.5 (HTx 64.7% vs. 52.9% (*p* = 0.464), recovery 17.6% vs. 5.9% (*p* = 0.292)), while LVAD implantation rates were similar (11.8% vs. 17.6%, *p* = 1.0). Major bleeding (17.6% vs. 47.1%, *p* = 0.141), stroke/TIA (5.9% vs. 17.6%, *p* = 0.601), and the need for renal replacement therapy (5.9% vs. 23.5%, *p* = 0.335) were numerically lower with Impella 5.5. **Conclusions:** In this single-center, retrospective analysis, the introduction of Impella 5.5 was associated with higher short-term survival and favorable bridging metrics; estimates are imprecise due to small, heterogeneous samples. These hypothesis-generating findings warrant confirmation in larger, prospective multicenter cohorts

## 1. Introduction

Cardiogenic shock (CS) remains one of the most challenging conditions in cardiovascular medicine and continues to be associated with unacceptably high mortality despite advances in reperfusion, critical care, and temporary mechanical circulatory support (MCS). Contemporary registries demonstrate that outcomes differ substantially by etiology: patients with acute myocardial infarction-related CS (AMI-CS) generally experience worse survival than those with decompensated chronic heart failure (HF-CS), even when advanced therapies are applied [1]. These differences are driven by distinct hemodynamic and metabolic trajectories, emphasizing the need for individualized treatment strategies guided by shock phenotype [2].

In many healthcare systems, care for CS is organized in a hub-and-spoke model, where delayed transfer from referring hospitals may affect outcomes and timely access to advanced therapies [3]. Traditional support strategies, such as the intra-aortic balloon pump (IABP), have not demonstrated consistent survival benefits in randomized trials [4]. Similarly, the ECLS-SHOCK trial found no mortality benefit of early veno-arterial extracorporeal membrane oxygenation (ECMO) in AMI-CS [5]. By contrast, the DanGer SHOCK trial reported improved survival with Impella CP compared with standard care in selected AMI-CS patients [6]. These findings underscore that the choice and timing of MCS devices critically influence outcomes.

The development of higher-capacity microaxial pumps, particularly the surgically implanted Impella 5.0 and 5.5, represents a major step forward. Impella 5.5 is capable of delivering flows up to 5–6 L/min, allows prolonged support through axillary access, and is associated with reduced hemolysis compared with smaller devices [7,8]. These features make it particularly attractive for patients with severe or refractory CS who require longer stabilization or multiorgan recovery.

Recent analyses from the Cardiogenic Shock Working Group (CSWG) registry have further advanced our understanding of patient selection and outcomes with microaxial devices. Carnicelli et al. demonstrated important etiologic differences in outcomes between AMI-CS and HF-CS patients supported with Impella CP, underscoring the heterogeneity of the shock population and the need for tailored device strategies [9]. Kanwar et al. analyzed outcomes of patients supported with Impella 5.5 for more than 14 days, reporting encouraging survival rates in a large real-world cohort and highlighting the feasibility of extended high-flow support [10]. In parallel, a 2025 consensus statement from the PeriOperative Quality Initiative and the Enhanced Recovery After Surgery (ERAS)-Cardiac Society outlined best-practice recommendations for temporary MCS, focusing on patient selection, escalation/de-escalation algorithms, and standardized reporting [11]. Together, these advances frame an evolving evidence base but also illustrate persistent gaps in knowledge.

Beyond classical shock stabilization, Impella 5.5 has been successfully applied in complex interventional and structural cardiology procedures requiring hemodynamic support. For example, it has been reported as a bridge strategy during transcatheter mitral valve repair in a patient with CS awaiting heart transplantation [12]. Such experiences emphasize the versatility of the device and support further investigation into its role across diverse clinical settings.

Importantly, while recent registry analyses have provided large-scale outcome data, there remains a lack of single-center, hypothesis-generating studies from Central and Eastern Europe. Moreover, most multicenter reports, although valuable, lack granularity regarding transfer pathways, device sequencing, and escalation strategies that are highly relevant to daily practice. Our group has previously described the establishment and early outcomes of the first dedicated Shock Team in Poland [13], which provides the organizational background for the present study. To our knowledge, this is the first report from the region evaluating Impella 5.5 with propensity-matched comparison to a historical cohort, capturing center-specific treatment patterns within a hub-and-spoke model.

Against this backdrop, we aimed to evaluate whether the introduction of Impella 5.5 in our center—either as stand-alone therapy or in combination with other MCS devices—was associated with improved survival compared with a historical cohort managed without Impella 5.5. Using propensity score matching on clinically salient variables, we sought to create balanced groups and provide an exploratory, hypothesis-generating estimate of short-term and six-month outcomes in a real-world single-center experience. Given the single-center and retrospective nature of our work, the present analysis was designed as a hypothesis-generating evaluation of outcomes after the introduction of Impella 5.5 within an institutional Shock Team program.

## 2. Materials and Methods

### 2.1. Patients Selection/Data Collection

This was a retrospective single-center study conducted at the University Hospital in Wroclaw, a tertiary referral “hub” institution providing advanced cardiac surgery (including heart transplantation [HTx] and durable LVAD implantation), interventional cardiology, and intensive care services. The hospital operates within a hub-and-spoke system, with many patients transferred from regional hospitals for escalation of therapy.

Only patients with cardiogenic shock who received temporary mechanical circulatory support (MCS) were included. Patients managed without MCS, those supported during ongoing cardiac arrest, and patients older than 70 years, and patients with isolated right ventricular failure were excluded.

The aim of this study was to evaluate whether the introduction of Impella 5.5 into our institutional Shock Team program was associated with improved outcomes in patients with cardiogenic shock. Impella 5.5 was used both as stand-alone support and as adjunctive or sequential therapy in combination with other temporary MCS devices, reflecting real-world Shock Team practice. Some patients had multiple device strategies, such as initial support with one device followed by escalation to another.

Accordingly, we compared two cohorts:

1. Impella 5.5 group—patients treated after the implementation of Impella 5.5, either as solo therapy or in combination/sequentially with other MCS devices;
2. Historical control group—patients treated before Impella 5.5 was available who received other forms of MCS (ECMO, Impella CP, CentriMag, IABP), either as stand-alone devices or in sequential/combined strategies, according to the Shock Team practice of that era.

The study protocol was approved by the Institutional Ethics Committee of Wroclaw Medical University (approval no. KB 43/2024), which waived the requirement for informed consent due to the retrospective and deidentified nature of the analysis.

#### 2.1.1. Propensity Score Matching

To assess the robustness of our findings, patients treated with Impella 5.5 were compared with a propensity score-matched control group. Propensity scores were estimated using logistic regression based on baseline covariates including age, sex, etiology of shock (ischemic vs. non-ischemic), serum lactate concentration at initiation of MCS, and SCAI shock stage (C–D grouped together vs. E). Matching was performed using the nearest-neighbor method without replacement and a caliper of 0.2 standard deviations of the logit of the propensity score.

Covariate balance was evaluated using standardized mean differences (SMDs), with values < 0.1 considered indicative of adequate balance. After matching, balance improved substantially and most covariates met the <0.1 threshold; however, sex remained above the threshold due to the very small number of female patients in the overall cohort. Propensity score distributions before and after matching were visualized with density plots, and overall balance was illustrated with a Love plot (Supplementary Figures S1 and S2).

#### 2.1.2. Outcomes

The primary outcome was survival at hospital discharge, 30 days, 3 months, and 6 months.

Secondary outcomes focused on the ability of MCS to bridge patients to a definitive therapy, as follows:

1. Native recovery (weaning from temporary MCS without HTx or durable LVAD);
2. Heart transplantation (HTx);
3. Durable LVAD implantation (HeartMate 3).

Complications were defined as follows:

1. Neurological events: transient ischemic attack (TIA) or ischemic/hemorrhagic stroke confirmed by neurological assessment and neuroimaging;
2. Bleeding: events requiring surgical intervention or transfusion, classified according to Bleeding Academic Research Consortium (BARC) criteria where applicable;
3. Peripheral ischemia: clinically significant limb ischemia requiring intervention or resulting in amputation.

#### 2.1.3. Statistical Analysis

Continuous variables are presented as the mean ± standard deviation (SD) or median with interquartile range (IQR) and were compared using the Mann–Whitney U test. Categorical variables are expressed as counts and percentages, and were compared using chi-square or Fisher’s exact test.

Survival was analyzed with Kaplan–Meier curves and compared using the log-rank test. Cox proportional hazards models were used to estimate hazard ratios (HR) and 95% confidence intervals (CI) for mortality. All statistical tests were two-sided, and *p*-values < 0.05 were considered significant.

Analyses were performed using R software (version 4.4.0).

## 3. Results

### 3.1. Baseline Characteristics

A total of 57 patients with cardiogenic shock were included: 17 treated with Impella 5.5 and 40 managed with other MCS devices in the historical era. After propensity score matching, 34 patients were analyzed (17 per group).

Baseline characteristics are shown in Table 1. Before matching, patients in the Impella 5.5 group were older than those in the control cohort. Other prespecified covariates (sex, etiology, SCAI stage, lactate) also differed, reflecting the heterogeneity of the unmatched population. After matching, covariate balance improved substantially and most variables achieved acceptable thresholds (SMD < 0.1). However, sex remained imbalanced due to the very small proportion of female patients in the overall cohort. Detailed balance diagnostics are provided in Table 1.

### 3.2. Survival

**Figure 1:**
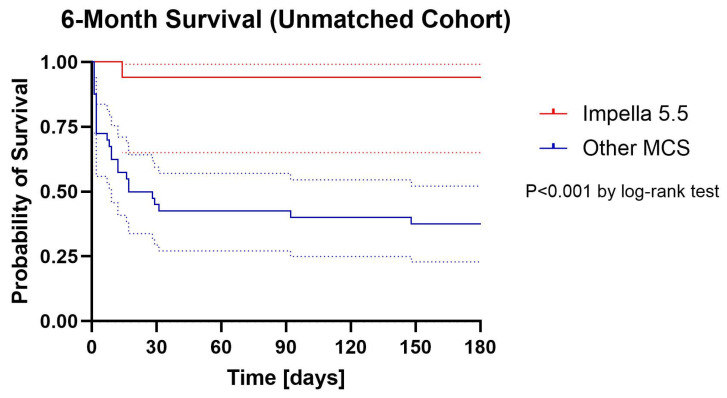
Cumulative Proportion Surviving (Kaplan–Meier) with 95% confidential intervals Unmatched Cohort. Dotted lines are 95% confidential intervals.

**Figure 2:**
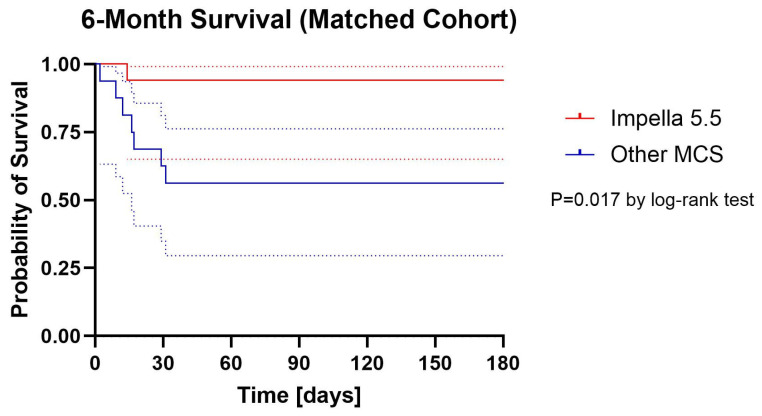
Cumulative Proportion Surviving (Kaplan–Meier) with 95% confidential intervals Matched Cohort. Dotted lines are 95% confidential intervals.

Kaplan–Meier survival curves are presented in Figure 1 (unmatched) and Figure 2. (matched). In the unmatched population, 6-month survival was significantly higher in the Impella 5.5 group compared with historical controls (88.2% vs. 37.5%, log-rank *p* < 0.001). In the matched cohort, 6-month survival remained numerically higher with Impella 5.5 (88.2% vs. 58.8%), and this difference reached statistical significance by log-rank analysis (*p* = 0.017).

In the matched cohort, survival to discharge (94.1% vs. 58.8%, *p* = 0.039), 30 days (94.1% vs. 58.8%, *p* = 0.039), and 3 months (94.1% vs. 58.8%, *p* = 0.039) was significantly higher in the Impella 5.5 group. At 6 months, survival remained numerically higher with Impella 5.5 (88.2% vs. 58.8%), but point analysis did not reach statistical significance (*p* = 0.118). However, Kaplan–Meier log-rank analysis of the matched cohort demonstrated a significant difference in overall survival between groups (*p* = 0.017).

On Cox regression, Impella 5.5 support was associated with a 93.7% reduction in the hazard of death in the unmatched cohort (HR 0.063, 95% CI 0.008–0.463, *p* = 0.007) and an 88.0% reduction in the matched cohort (HR 0.12, 95% CI 0.015–0.973, *p* = 0.047).

Absolute survival rates at discharge, 30 days, 3 months, and 6 months are summarized in Table 2. In both the unmatched and matched populations, survival to discharge, 30 days, and 3 months was significantly higher in the Impella 5.5 group, whereas the 6-month point estimate favored Impella 5.5 but did not reach statistical significance in matched analysis.

### 3.3. Bridging to Definitive Therapy

Bridging outcomes are summarized in Table 2. In the unmatched cohort, Impella 5.5 was associated with significantly higher odds of successful bridging (to recovery, HTx, or LVAD implantation) compared with controls (OR 11.826, 95% CI 1.426–98.062, *p* = 0.022). In the matched cohort, the association remained directionally consistent but was not statistically significant (OR 6.667, 95% CI 0.686–64.771, *p* = 0.102).

When analyzed by type of definitive therapy, no statistically significant differences were observed between groups. However, trends favored Impella 5.5, with more frequent bridging to heart transplantation (64.7% vs. 52.9% in matched controls) and native recovery (17.6% vs. 5.9%), while rates of durable LVAD implantation (HeartMate 3) were similar between groups (11.8% vs. 17.6%). These findings suggest that the introduction of Impella 5.5 may facilitate bridging to recovery or transplantation in selected patients, although confirmation requires larger cohorts.

### 3.4. Complications

Bleeding events of BARC grade ≥3a occurred in 17.6% of patients supported with Impella 5.5 compared to 47.1% of matched controls (OR 0.23, 95% CI 0.05–1.16, *p* = 0.14). When analyzed separately, both BARC 3a (5.9% vs. 23.5%) and BARC 3b events (11.8% vs. 23.5%) tended to be less frequent in the Impella 5.5 group, although these differences were not statistically significant.

Neurological complications were also numerically less common with Impella 5.5, with stroke or TIA occurring in 5.9% compared to 17.6% of controls (OR 0.29, 95% CI 0.03–2.91, *p* = 0.60).

Renal replacement therapy was required in 5.9% of patients in the Impella 5.5 group and 23.5% of controls (OR 0.20, 95% CI 0.02–1.98, *p* = 0.34). Heparin-induced thrombocytopenia occurred with similar frequency in both groups (11.8% vs. 11.8%, OR 1.00, 95% CI 0.12–8.59, *p* = 1.00).

Overall, the use of Impella 5.5 was associated with numerically lower rates of major bleeding, neurological events, and renal replacement therapy, but the study was not powered to detect statistically significant differences. Rare complications such as limb ischemia, hemolysis, or insertion site infection were not observed or occurred in isolated case (Table 3).

## 4. Discussion

In this single-center, retrospective study, the introduction of Impella 5.5 into our Shock Team algorithm was associated with improved short-term survival compared with a historical cohort treated with other mechanical circulatory support (MCS) strategies. Survival at discharge, 30 days, and t 3 months was significantly higher in patients receiving Impella 5.5, whereas 6-month survival showed a favorable numerical trend, though not statistically significant in point analysis. Kaplan–Meier curves demonstrated a significant overall difference, suggesting a potential survival advantage with Impella 5.5 in this high-risk population.

Our results complement and extend the growing body of evidence on temporary MCS in cardiogenic shock (CS). The ECLS-SHOCK trial demonstrated no mortality benefit of early veno-arterial extracorporeal membrane oxygenation (ECMO) in AMI-CS [5], whereas the DanGer SHOCK trial showed improved outcomes with Impella CP compared with standard care in selected AMI-CS patients [6]. These data underscore the importance of device choice and timely escalation in determining outcomes. More recently, registry analyses from the Cardiogenic Shock Working Group (CSWG) have provided important insights. Carnicelli et al. reported outcome differences between AMI-CS and HF-CS patients supported with Impella CP [9], while Kanwar et al. showed favorable survival in patients maintained on Impella 5.5 for more than 14 days [10]. Our findings are directionally consistent with these studies, despite being derived from a smaller single-center cohort.

Compared with registry analyses, our study offers complementary granularity. Large registries provide statistical power and external validity but often lack detail on institutional protocols, transfer patterns, and device sequencing. In contrast, our experience reflects real-world practice in a Central European hub-and-spoke system, where patient transfer delays and resource availability strongly influence outcomes. This perspective is particularly relevant, as it mirrors the challenges faced by many medium-volume centers implementing advanced MCS programs. It also builds upon our previous report describing the establishment and early outcomes of the first dedicated Shock Team in Poland [13]. In that study, the use of MCS was identified as an independent predictor of improved in-hospital survival in multivariable analysis, underscoring the central role of advanced support strategies in the management of cardiogenic shock. The present analysis extends these observations by specifically evaluating the introduction of Impella 5.5 into the Shock Team algorithm, highlighting how its integration may further improve outcomes.

Beyond classical shock stabilization, Impella 5.5 has also been successfully applied in complex structural interventions in critically ill patients. In particular, it has been reported as a bridge strategy during transcatheter mitral valve repair in a patient with CS awaiting transplantation [12], and, more recently, in combination with LVAD support for acute ischemic mitral regurgitation–related shock [14]. These experiences emphasize the versatility of Impella-supported mitral interventions and illustrate how integration of high-flow devices can expand therapeutic options for otherwise inoperable patients. Moreover, its emerging role as an adjunct during complex ventricular tachycardia ablation underscores the potential of high-flow percutaneous support beyond traditional shock management [15].

Beyond these findings, our study highlights important future directions. Larger multicenter cohorts and randomized controlled trials are needed to confirm the survival benefit of Impella 5.5 and to define optimal timing, patient selection, and integration with other MCS devices. Particular emphasis should be placed on refining Shock Team algorithms, identifying clinical or biomarker-based predictors of response, and harmonizing complication reporting in line with international consensus recommendations [11]. Clinically, the introduction of Impella 5.5 into a hub-and-spoke system demonstrates how adoption of higher-capacity microaxial pumps can shift treatment paradigms even in medium-volume centers. These results may therefore inform not only tertiary referral hospitals but also regional networks developing advanced MCS programs.

Taken together, these results from a single institution suggest a possible short-term survival advantage associated with Impella 5.5. However, given the retrospective design, small sample size and heterogeneity, the data should be interpreted strictly as hypothesis-generating and not definitive.

### Study Limitations

This study has several important limitations. First, it was a retrospective, single-center analysis with a relatively small and heterogeneous cohort, which limits the generalizability of the findings. Although propensity score matching was applied to minimize baseline imbalances, residual confounding cannot be excluded, and balance was not perfect for some variables such as sex. Second, detailed hemodynamic data and lactate trajectories were incomplete, precluding a more granular evaluation of physiological response to support. Finally, the small sample size limits the statistical power, particularly for secondary outcomes and complications. Therefore, the results should be considered hypothesis-generating rather than definitive, serving as a starting point for larger, prospective, multicenter studies.

## 5. Conclusions

The introduction of Impella 5.5 into our institutional Shock Team program—used either as stand-alone support or in combination with other MCS devices—was associated with improved short-term survival in patients with severe cardiogenic shock compared with a historical cohort. Six-month survival showed a favorable trend, although not statistically significant in point analysis.

These findings suggest that incorporating Impella 5.5 into structured shock management algorithms may provide a meaningful clinical benefit, particularly in hub-and-spoke systems where timely escalation is essential. However, given the retrospective design, small sample size, and residual heterogeneity, the results should be considered hypothesis-generating rather than definitive. Larger prospective multicenter studies are required to validate these observations and to better define the role of Impella 5.5 within contemporary Shock Team practice.

## Figures and Tables

**Table 1:** Patient characteristics.

Variable	All Patients				Propensity-Matched Groups			
	Impella 5.5 Group (N = 17)	Control Group (N = 40)	*p*	SMD	Impella 5.5 Group (N = 17)	Control Group (N = 17)	*p*	SMD
Age, y, mean (SD)	48.71 (11.12)	52.38 (12.52)	0.179	−0.379	48.71 (11.12)	46 (11.37)	0.569	0.243
Female gender, n (%)	1 (5.88%)	7 (17.50%)	0.413	0.439	1 (5.88%)	3 (17.65%)	0.601	0.5
Ischemic etiology of shock, n (%)	8 (47.06%)	22 (55.00%)	0.795	−0.194	8 (47.06%)	7 (41.18%)	1	0.118
SCAI E, n (%)	4 (23.53%)	13 (32.50%)	0.718	0.274	4 (23.53%)	3 (17.65%)	1	−0.139
Lactate, mmol/L, mean (SD)	3.46 (3.45)	4.79 (3.91)	0.136	−0.385	3.46 (3.45)	3.58 (2.53)	0.408	−0.034
Hypertension, n (%)	6 (35.29%)	24 (60.00%)	0.156	unmatched	6 (35.29%)	9 (52.94%)	0.49	unmatched
Diabetes, n (%)	3 (17.65%)	6 (15.00%)	1	unmatched	3 (17.65%)	2 (11.76%)	1	unmatched
Coronary artery disease, n (%)	6 (35.29%)	17 (42.50%)	0.832	unmatched	6 (35.29%)	5 (29.41%)	1	unmatched
Stroke/TIA, n (%)	0 (0.00%)	3 (7.50%)	0.547	unmatched	0 (0.00%)	2 (11.76%)	0.485	unmatched

Abbreviations: SMD, standardized mean difference; SCAI, Society for Cardiovascular Angiography & Interventions; SD, standard deviation; TIA, transient ischemic attack.

**Table 2:** Outcomes.

Outcome	All Patients			Propensity-Matched Groups		
	Impella 5.5 Group (N = 17)	Control Group (N = 40)	*p*	Impella 5.5 Group (N = 17)	Control Group (N = 17)	*p*
Survival—Discharge	94.1% (16/17)	40% (16/40)	0.001	94.1% (16/17)	58.8% (10/17)	0.039
Survival—30 days	94.1% (16/17)	40% (16/40)	0.001	94.1% (16/17)	58.8% (10/17)	0.039
Survival—3 months	94.1% (16/17)	40% (16/40)	0.001	94.1% (16/17)	58.8% (10/17)	0.039
Survival—6 months	88.2% (15/17)	37.5% (15/40)	0.001	88.2% (15/17)	58.8% (10/17)	0.118
Recovery	17.6% (3/17)	7.5% (3/40)	0.349	17.6% (3/17)	5.9% (1/17)	0.292
Heart transplantation	64.7% (11/17)	40.0% (16/40)	0.156	64.7% (11/17)	52.9% (9/17)	0.464
Durable LVAD (HM3)	11.8% (2/17)	12.5% (5/40)	1.000	11.8% (2/17)	17.6% (3/17)	1.000

Abbreviations: LVAD (HM3), left ventricular assist device HertMate 3.

**Table 3:** Major complications.

Outcome	Impella 5.5 Group (N = 17) 95% CI	Matched Controls Group (N = 17) 95% CI	Odds Ratio (95% CI)	*p*
BARC ≥3a bleeding	17.6% (3/17) 3.8–43.4%	47.1% (8/17) 23.0–72.2%	0.24 (0.05–1.16)	0.141
Stroke/TIA	5.9% (1/17) 0.1–28.7%	17.6% (3/17) 3.8–43.4%	0.29 (0.03–3.13)	0.601
Renal replacement therapy	5.9% (1/17) 0.1–28.7%	23.5% (4/17) 6.8–49.9%	0.20 (0.02–2.05)	0.335
HIT	11.8% (2/17) 1.5–36.4%	11.8% (2/17) 1.5–36.4%	1.00 (0.12–8.06)	1.000

Abbreviations: CI = confidence interval. Odds ratios with 95% CIs were derived from 2 × 2 contingency tables; *p*-values from two-sided Fisher’s exact test. No correction for multiple comparisons was applied given the exploratory nature of the analysis.

## Data Availability

The raw data supporting the conclusions of this study will be made available by the authors on request.

## Supplementary Materials

The following supporting information can be downloaded at: https://www.mdpi.com/article/10.3390/jcm14217552/s1, Figure S1: Propensity Score-Matching Love Plot; Figure S2: Propensity Score-Matching Density Plot.
