# Supplementary figures and images for: The Introduction of Impella 5.5 in Cardiogenic Shock: A Single-Center, Retrospective Propensity Score-Matched Analysis
Source: J Clin Med. 2025 Oct 24;14(21):7552. doi: 10.3390/jcm14217552 (PMC12610547; doi:10.3390/jcm14217552)

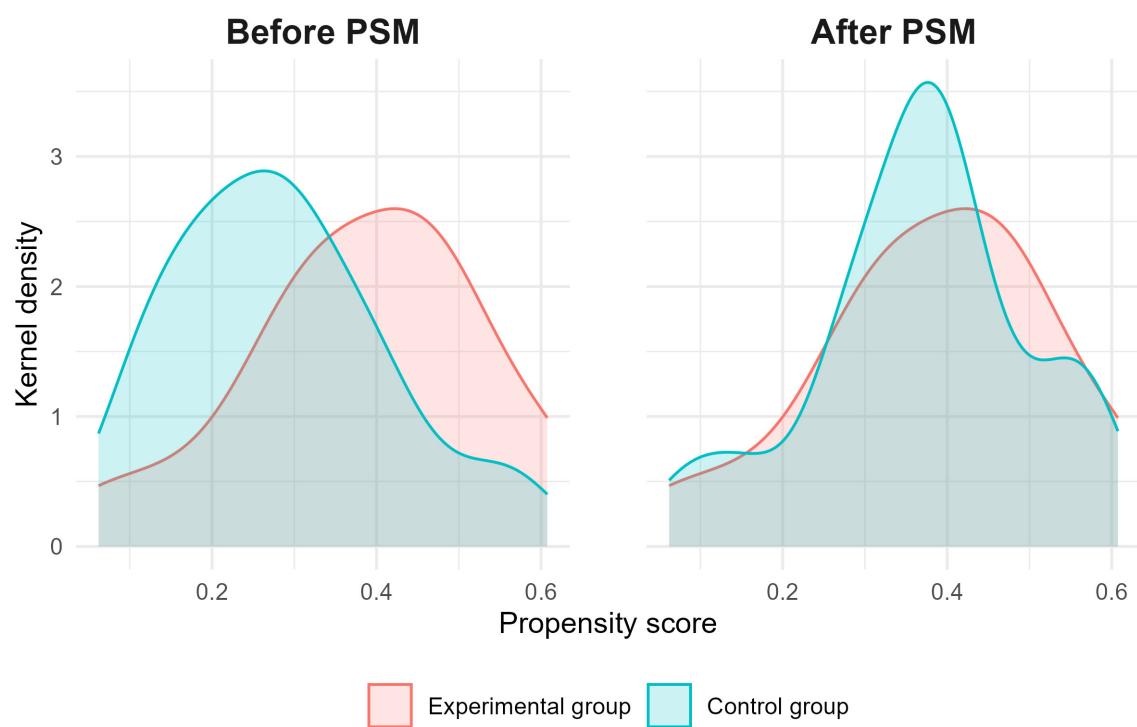

**Figure S1.** Propensity Score-Matching Love Plot.

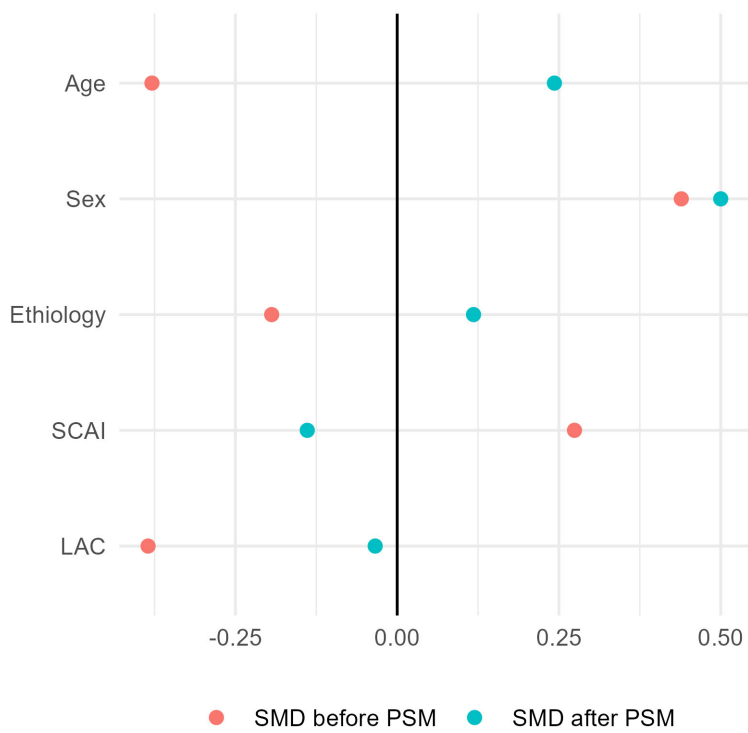

**Figure S2.** Propensity Score-Matching Density Plot.

Supplement: Supplementary file 1 [file jcm-14-07552-s001.zip › jcm-3868118-supplementary.pdf]
